# RETRACTED: Alhakamy et al. Ceftriaxone and Melittin Synergistically Promote Wound Healing in Diabetic Rats. *Pharmaceutics* 2021, *13,* 1622

**DOI:** 10.3390/pharmaceutics16091168

**Published:** 2024-09-05

**Authors:** Nabil A. Alhakamy, Giuseppe Caruso, Basma G. Eid, Usama A. Fahmy, Osama A. A. Ahmed, Ashraf B. Abdel-Naim, Abdulmohsin J. Alamoudi, Shareefa A. Alghamdi, Hadeel Al Sadoun, Basmah M. Eldakhakhny, Filippo Caraci, Wesam H. Abdulaal

**Affiliations:** 1 Department of Pharmaceutics, Faculty of Pharmacy, King Abdulaziz University, Jeddah 21589, Saudi Arabia; nalhakamy@kau.edu.sa (N.A.A.); uahmedkauedu.sa@kau.edu.sa (U.A.F.); oaahmed@kau.edu.sa (O.A.A.A.); 2 Center of Excellence for Drug Research and Pharmaceutical Industries, King Abdulaziz University, Jeddah 21589, Saudi Arabia; ajmalamoudi@kau.edu.sa; 3 Mohamed Saeed Tamer Chair for Pharmaceutical Industries, King Abdulaziz University, Jeddah 21589, Saudi Arabia; 4 King Fahd Medical Research Center, King Abdulaziz University, Jeddah 21589, Saudi Arabia; 5 Department of Drug and Health Sciences, University of Catania, 95125 Catania, Italy; 6 Department of Pharmacology and Toxicology, Faculty of Pharmacy, King Abdulaziz University, Jeddah 21589, Saudi Arabia; beid@kau.edu.sa (B.G.E.); aaabdulalrahman1@kau.edu.sa (A.B.A.-N.); 7 Department of Biochemistry, Faculty of Science, Cancer and Mutagenesis Unit, King Fahd Medical Research Center, King Abdulaziz University, Jeddah 21589, Saudi Arabia; saaalghamdi1@kau.edu.sa (S.A.A.); whabdulaal@kau.edu.sa (W.H.A.); 8 Department of Medical Laboratory Technology, Faculty of Applied Medical Sciences, King Abdulaziz University, Jeddah 21589, Saudi Arabia; hsadounkau.ed.sa@kau.edu.sa; 9 Department of Clinical Biochemistry, Faculty of Medicine, King Abdulaziz University, Jeddah 21589, Saudi Arabia; beldakhakhny@kau.edu.sa; 10 Oasi Research Institute—IRCCS, 94018 Troina, Italy

The journal retracts the article, “Ceftriaxone and Melittin Synergistically Promote Wound Healing in Diabetic Rats” [1]. 

Following publication, concerns were brought to the attention of the publisher regarding the overlap of images.

Adhering to our complaints procedure, an investigation was conducted that confirmed the duplication in Figure 2A, presenting different experimental conditions.

While the authors fully cooperated with the Editorial Office during the investigation, they were unable to satisfactorily explain the overlap of the figures and meet the required quality standards for raw images in order to warrant a correction as per the journal’s original image requirements policy (https://www.mdpi.com/journal/pharmaceutics/instruc-tions#oriimages). As a result, the Editorial Board and Editor-in-Chief were unable to confirm the reliability of the findings and subsequently decided to retract this paper.

This retraction was approved by the Editor-in-Chief of the journal *Pharmaceutics*. 

The authors did not agree to this retraction.
